# Elucidating the Reduction Mechanism of Lithium Bis(oxalato)borate

**DOI:** 10.1021/acs.jpclett.4c00328

**Published:** 2024-02-28

**Authors:** Tim Melin, Robin Lundström, Erik J. Berg

**Affiliations:** Department of Chemistry, Ångström Laboratory, Uppsala University, Box 538, SE-751 21 Uppsala, Sweden

## Abstract

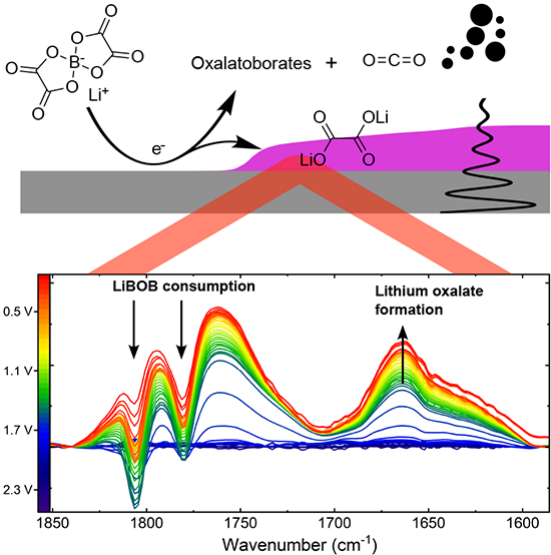

Electrolyte additives are indispensable to enhance the performance of Li-ion batteries. Lithium bis(oxalato)borate (LiBOB) has been explored for many years, as it improves both cathode and anode performance. No consensus regarding its reaction mechanisms has, however, been established. A model *operando* study combining attenuated total reflection infrared spectroscopy (ATR-FTIR), electrochemical quartz crystal microbalance (EQCM), and online electrochemical mass spectrometry (OEMS) is herein presented to elucidate LiBOB reduction and electrode/electrolyte interphases thus formed. Reduction of the BOB^–^ ion sets in at ∼1.8 V with solid lithium oxalate and soluble oxalatoborates as the main products. The reduced BOB^–^ ion also reacts with itself and its environment to evolve CO_2_, which in turn impacts the interphase formed on the negative electrode. This study provides further insights into the reduction pathways of LiBOB and how they contribute to the interphase formation.

Optimizing electrolyte composition is paramount for advancing Li-ion batteries. Additives are commonly introduced to fulfill the various functions necessary for Li-ion cell chemistry. Three main categories of additives are electrode/electrolyte interphase formers (on both anode and cathode), harmful species scavengers, and stabilizer agents.^1^ Additives are typically soluble in the electrolyte and come in the form of either a molecule or a salt. Lithium bis(oxalato)borate (LiBOB, Figure S1 and to the left in Figure 4) has garnered tremendous attention for its promising layer-forming properties on not only the anode but also the cathode.^2,3^ LiBOB was synthesized and patented in 1999 by Lischka et al.^4^ and has been studied extensively by Xu et al.^5−11^ among many others. One aspect of LiBOB that differs from many other additives is its relatively high reduction potential. Most layer-formers are being reduced at the anode at potentials close to the reduction potential of the electrolyte solvent ethylene carbonate (EC) < 0.9 V.^1^ LiBOB on the other hand, is reported to be reduced from 1.8–1.0 V,^10,12−14^ well before EC and many other additives, such as the well-known vinylene carbonate and fluoroethylene carbonate.^1^ Several reaction pathways have been proposed for the reduction of LiBOB, but most are similar and suggest the formation of lithium oxalate (Li_2_C_2_O_4_) and carbon dioxide (CO_2_) as the main reaction products. Apart from pure Li_2_C_2_O_4_, more complex molecular combinations of oxalate borates and boron-containing semicarbonates have been proposed. Reduced BOB^–^ species have also been suggested to react with carbonate solvents (e.g., EC) to form boron-containing semicarbonates^13^ and oligomers/polymers.^14^ The synthesis of LiBOB has shown to be somewhat challenging, and boron-containing impurities from synthesis have also been claimed to be the cause for the reduction at high potentials (>1.7 V).^15,16^

The primary focus of the study presented herein is to elucidate the reduction pathway of the BOB^–^ anion and comprehend the properties of the solid electrolyte interphase (SEI) thus formed in the process. Important is the effectiveness of the LiBOB-derived SEI in suppressing electrolyte degradation and accommodating the negative influence of typical contaminants like water. Most studies conducted to date on LiBOB performed *ex situ* or *post-mortem* chemical analysis of the SEI after Li-ion cell formation and/or after prolonged cycling by subjecting the electrode extracted from the cell to various spectroscopic (e.g., X-ray photoelectrode spectroscopy, vibrational spectroscopy)^7,9,10,12,14,17,18^ or microscopic (e.g., transmission electron microscopy)^13^ techniques. SEI formation is, however, well-recognized to be a complex multistep process, hardly captured by *ex situ* analytical approaches. Therefore, a set of complementary *operando* characterization techniques, namely, attenuated total reflection infrared spectroscopy (ATR-FTIR), electrochemical quartz crystal microbalance (EQCM), and online electrochemical mass spectrometry (OEMS), are here applied to monitor solid, liquid, and volatile reaction products, respectively, associated with LiBOB reduction during operation of the cell.

Figure 1 shows the results from operando ATR-FTIR of a 50 mM LiBOB containing a model electrolyte. Compared with a classic Li-ion electrolyte, the cathodically more stable LiClO_4_ salt dissolved in DME was applied to avoid the complexity of organic carbonate solvent decomposition and the extensive side-reactions of LiPF_6_. In order to ensure the purity of the LiBOB salt used herein, ^11^B-NMR was performed on pure salt dissolved in DMSO, and only one signal was observed and assigned to LiBOB (Figure S2). A significant influence from impurities remaining after the LiBOB synthesis is therefore unlikely. A porous glassy carbon (GC) composite working electrode was applied as an electrode to mimic the surface of the typical graphite Li-ion active material, but at the same time avoid effects associated with cosolvent Li^+^ intercalation. As the GC electrode was pressed against an ATR crystal, both the electrode surface and the electrolyte phase were probed. During linear sweep voltammetry (LSV), all spectra recorded were found to be dominated by vibrations from various electrolyte species (OCP spectrum in Figure 1a and Figure S3). Therefore, differential absorbance spectra (relative to a spectrum at the OCP) are presented in Figure 1b to amplify spectral changes. The current recorded during LSV from the OCP to 0.5 V (Figure 1c) shows a clear reduction peak setting in at 1.8 V associated with BOB^–^. Eleven spectral features (vertical dashed lines in Figure 1a,b) are identified, and their respective intensities are tracked as a function of electrode potential (Figure 1d).

**Figure 1:**
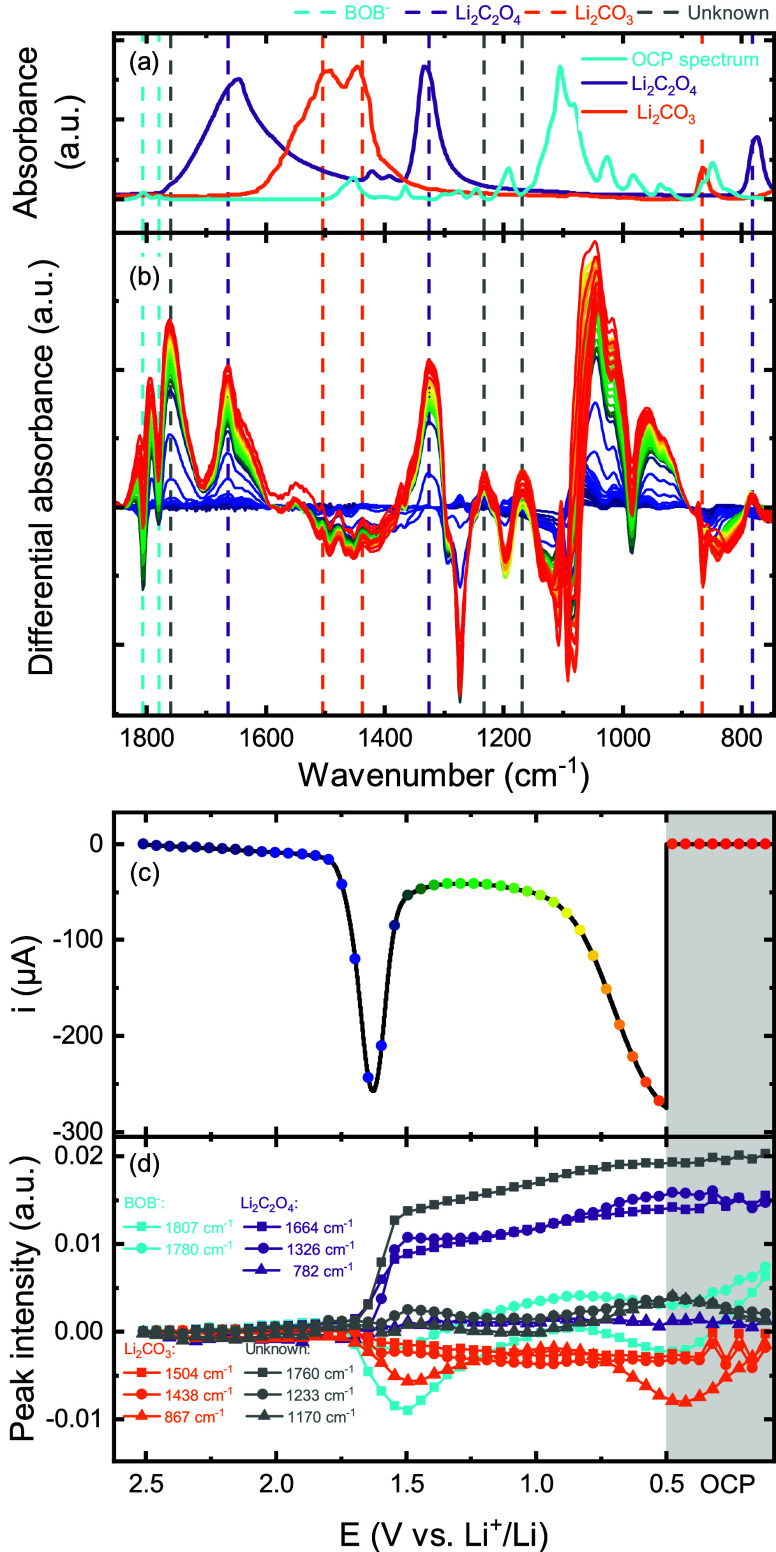
(a) Reference absorbance spectra of a porous GC soaked in 0.2 M LiClO_4_ in DME + 50 mM LiBOB at OCP (“OCP spectrum” in figure), Li_2_C_2_O_4_, and Li_2_CO_3_, (b) *operando* IR differential absorbance spectra relative to a spectrum at open-circuit potential (OCP) with corresponding (c) linear sweep voltammogram of a porous GC electrode pressed on a diamond ATR crystal in 0.2 M LiClO_4_ in DME + 50 mM LiBOB and (d) peak intensities of selected wavenumbers as a function of electrode potential. Spectral color coding in panel b corresponds to data points in panel c.

The spectra remain more or less unchanged from those of the OCP until the reduction current sets in at 1.8 V, except for minor changes in the ratio between Li^+^-coordinated and free DME, likely as a consequence of Li^+^ adsorption to GC during the negative polarization. Both positive and negative going peak intensities appear <1.8 V and are related to vibrational bands from newly formed or consumed species, respectively. A majority of positive peaks pair with one or multiple negative peaks and are simply attributed to changes in the ratio between free and Li^+^-coordinated DME. For instance, a set of peaks found in the range of 900–1150 cm^–1^ are dominated by the C–O vibration modes of free and coordinated DME, respectively. Deconvoluting these changes in the electrolyte from emerging bands from new species is limited by the breadth and overlap of these features. Even though this is the case for the majority of positive peaks, a few new peaks with little to no overlap with DME can be analyzed. Two peaks at 1807 and 1780 cm^–1^ are assigned to valence vibrations of the C=O bond in BOB^–^ (in accordance with the literature^19^) and display a rapid drop in intensity <1.8 V, which shows that BOB^–^ is being consumed in the reduction process (Figure 1d). When that reduction process ends <1.5 V the intensity of the same peaks increases due to replenishment of BOB^–^ lost at the electrode surface from the bulk electrolyte, thus implying that only a minor fraction of BOB^–^ at the electrode surface undergoes reduction. A second intensity decrease for BOB^–^ sets in <0.8 V, but now as a result of the consumption of Li^+^ ions due to its stronger adsorption on the GC electrode. After the LSV, the intensity associated with BOB^–^ increases continuously throughout the whole OCP step, reaching even higher intensities than in the OCP spectrum. During BOB^–^ reduction, three strong peaks emerge at 1664, 1326, and 780 cm^–1^, which agree with the spectrum of Li_2_C_2_O_4_. These peaks grow fast during reduction, but they slow down <1.5 V when no reductive current flows and remain unchanged during the OCP step; that is, no conversion or dissolution of Li_2_C_2_O_4_ is observed. Along with the formation of Li_2_C_2_O_4_, another set of peaks emerges at 1504, 1438, and 867 cm^–1^, which are assigned to Li_2_CO_3_. However, these features are very small and overlap with the spectra of the OCP and are therefore not considered to be a major reduction product of BOB^–^. The intensity of 867 cm^–1^ does not follow the same trend as the other two but resembles the profile of the BOB^–^ vibration, demonstrating the difficulties induced by spectral overlaps. Three additional peaks emerge <1.8 V at 1760, 1233, and 1170 cm^–1^ and follow the same trend as the Li_2_C_2_O_4_ vibrations, hence suggesting that these three peaks are associated with the remaining oxalatoborates from BOB^–^ reduction, as outlined below. The interaction between reduced BOB^–^ and EC was studied with operando FTIR by assembling cells with 0.2 M LiClO_4_ in DME + 50 mM LiBOB + 5 vol % EC as electrolyte. Unfortunately, no clear additional features besides the ones already discussed in the sections above could be deconvoluted (Figure S4).

Figure 2 shows the cyclic voltammograms along with the corresponding mass change *Δm* and calculated mass per electron (mpe, grams of deposit per mol electrons) value of carbon-coated QCM sensors cycled in the DME electrolyte including no additive and 50 mM LiBOB. Neither current peaks nor significant mass deposition were recorded when LIBOB was absent, hence confirming the cathodic stability of LiClO_4_ in DME. The voltammogram for the LiBOB containing electrolyte is similar to the IR cell (Figure 1c) containing the same electrolytes with a reduction peak attributed to BOB^–^ reduction starting at 1.8 V. The current <1 V is lower for LiBOB, which is related to the higher cell impedance induced by the LiBOB-derived SEI formed >1 V. Indeed, *Δm* increases along with the reduction current and slows down as the current decays. At the same time, the highest mpe value of 50 g mol^–1^ is recorded for the LiBOB electrolyte at the peak in the voltammogram, which again suggests the formation of Li_2_C_2_O_4_ possessing a mpe-value of 51 g mol^–1^ (assuming a two-electron per Li_2_C_2_O_4_ process). An increase in *Δm* is observed <1 V with essentially no current flow, except for the adsorption/desorption of Li^+^ ions. Again, as noted in the ATR-FTIR experiment above, no electrochemical conversion of lithium oxalate is likely, but rather other chemical reactions possibly involve metastable oxalatoborates and the remaining LiBOB salt.

**Figure 2:**
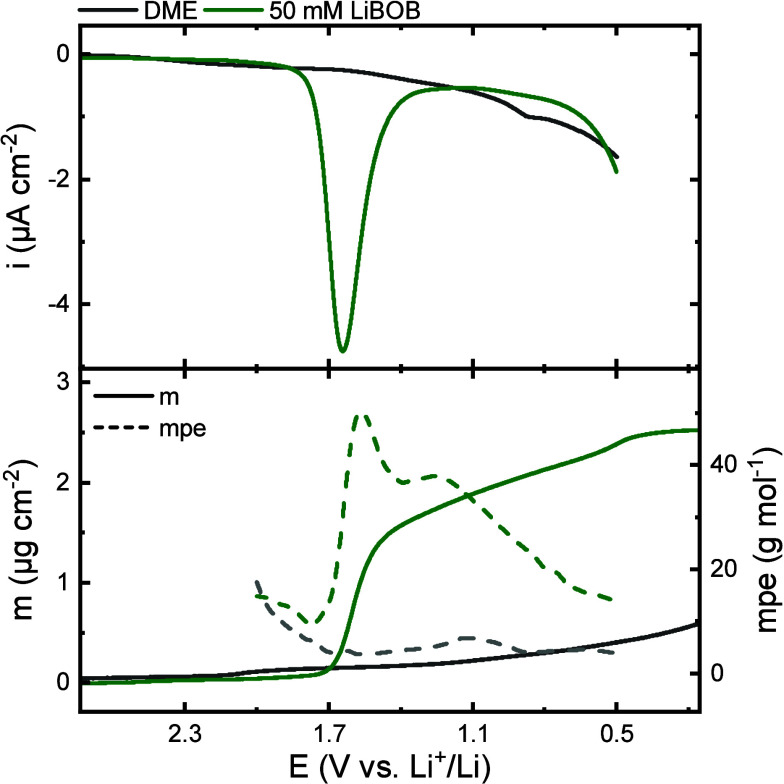
Cyclic voltammograms of carbon-coated QCM sensors in 0.2 M LiClO_4_ in DME with and without 50 mM LiBOB with corresponding mass deposition on the sensors and calculated mpe values during the negative sweep.

Figure 3 shows cyclic voltammograms (starting from the OCP and vertex potentials 0.5 and 1.7 V) and associated CO_2_, C_2_H_4_, and H_2_ gas evolution profiles for GC electrodes cycled in the baseline electrolyte, 50 mM LiBOB, 5% EC, as well as 50 mM LiBOB + 5% EC. EC was added in order to monitor LiBOB reactivity toward EC and its ability to suppress EC reduction <0.9 V.^1^ Again, a reduction peak starting at 1.8 V is observed when LiBOB is present but now along with the evolution of CO_2_. The total amount of CO_2_ evolved is 0.85 nmol cm^–2^ (for 50 mM LiBOB), which is significantly lower than the charge consumed (24.3 nmol electrons cm^–2^), hence demonstrating that the CO_2_ evolution is not a major product of the electrochemical reduction of LiBOB. The addition of LiBOB and generation of CO_2_ further suppress H_2_ evolution. H_2_ is well-known to derive from the reduction of water impurities, but the reduced protons can be scavenged in the presence of CO_2_ to form Li-formate rather than inducing hydrogen evolution reaction.^20^ Compared to LiBOB, the decomposition of EC is much less efficient in generating CO_2_ and H_2_ evolves similarly to when no additive is present. As expected, EC reduction results in C_2_H_4_ evolution <1 V. When both LiBOB and EC are present (orange, Figure 3), LiBOB is reduced before EC, which suppresses not only H_2_ evolution but also the reduction of EC. Interestingly, more CO_2_ evolves when both LiBOB and EC are present, which likely is a result of ring-opening of EC by the reduced BOB^–^ products. Although the cells display higher impedance, as judged from the magnitude of the reversible currents (Figure 3), the LiBOB-derived SEI on the carbon electrode surface is more passivated and effectively blocks the reduction of both the EC and impurities. This points to the direction that LiBOB provides faster and more effective passivation of the carbon anode compared to EC.

**Figure 3:**
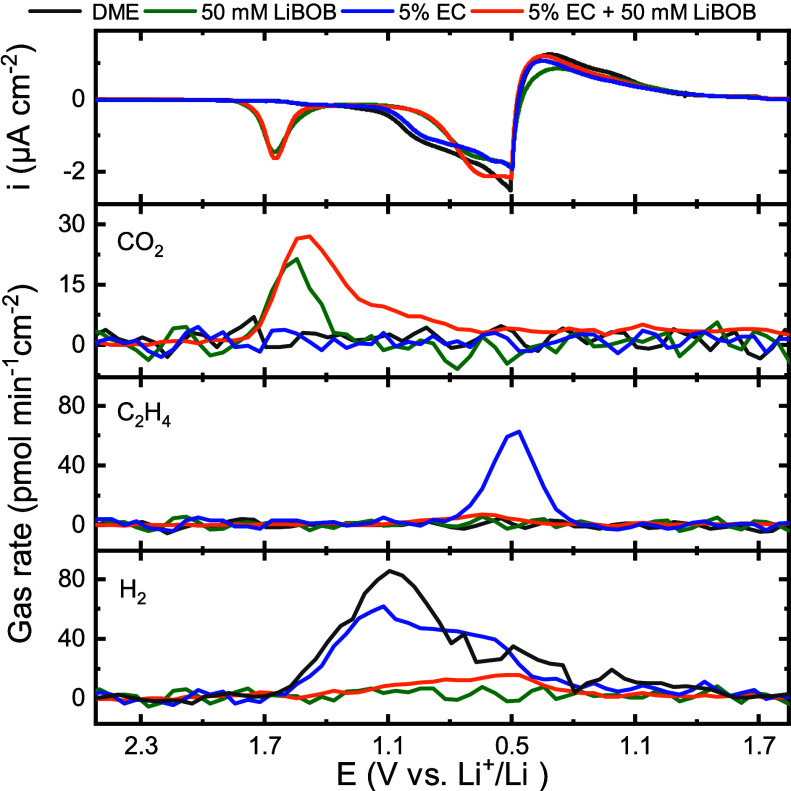
Cyclic voltammograms of GC porous electrodes in 0.2 M LiClO_4_ in DME with LiBOB and EC added with corresponding gas evolution rates of CO_2_, C_2_H_4_, and H_2_.

A model study of the electrochemical reduction mechanism of LiBOB, its SEI-forming ability, and chemical side-reactions toward itself and EC is presented and illustrated in Figure 4. A clear reduction peak <1.8 V is observed in all cells containing LiBOB and associated with the formation of an SEI primarily based on Li-oxalate. LiBOB reduction should also result in oxalatoborates along with minor amounts of Li_2_CO_3_ and CO_2_ from subsequent chemical side-reactions between reduced BOB^–^ species and the rest of the electrolyte. Li_2_CO_3_ may however likely stem from water impurity reduction and generation of LiOH, which in turn reacts with the evolving CO_2_. LiBOB is an efficient SEI former, which in spite of a higher cell impedance efficiently suppresses further electrolyte reduction and associated gas evolution. The fundamental insights into reaction pathways of LiBOB and other electrolyte additives are critical to deepen our understanding, advance electrolyte modeling, and accelerate the development of future battery electrolytes. In this study, complementary *operando* techniques are utilized to monitor solid, liquid, and gaseous reaction species during the reduction process of a crucial additive. By doing so, existing uncertainties surrounding the reduction mechanism of LiBOB are addressed. This study not only contributes to fundamental knowledge but also underscores the significance of advancing *operando* techniques. Furthermore, this demonstrates the value of revisiting well-studied systems with novel experimental approaches.

**Figure 4:**
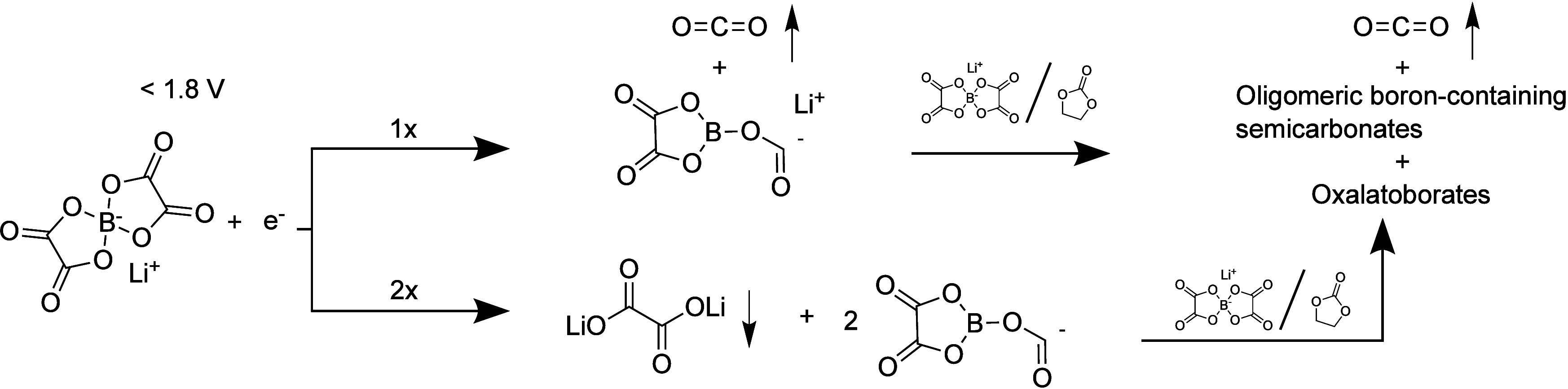
Schematic of two of the possible reduction mechanisms of LiBOB of which products could be identified with OEMS (CO_2_, top) and IR + EQCM (Li_2_C_2_O_4_, bottom). The arrows pointing up and down indicate gaseous and precipitating species, respectively.

## Supporting Information Available

Experimental details and 4 figures: structure of LiBOB (Figure S1), ^11^B-NMR of LiBOB salt (Figure S2), *operando* IR of 0.2 M LiClO_4_ in DME + 50 mM LiBOB (before subtraction of OCP spectrum, Figure S3), and +50 mM LiBOB + 5% EC (Figure S4) (PDF)
Transparent Peer Review report available (PDF)
