# Supplementary material for: Elucidating the Reduction Mechanism of Lithium Bis(oxalato)borate
Source: J Phys Chem Lett. 2024 Feb 28;15(9):2537–41. doi: 10.1021/acs.jpclett.4c00328 (PMC10926156; doi:10.1021/acs.jpclett.4c00328)
Supplement: Supplementary file 1 — jz4c00328_si_001.pdf [file jz4c00328_si_001.pdf]

# Supporting information: Elucidating the Reduction Mechanism of Lithium bis(oxalato)borate

*Tim Melin*, Robin Lundström, Erik J. Berg**

Department of Chemistry, Ångström Laboratory, Uppsala University, Box 538, SE-751 21 Uppsala, Sweden

*tim.melin@kemi.uu.se, erik.berg@kemi.uu.se

## 1. Experimental

1,2-methoxyethane, DME, (CHROMASOLV, 99.9%, inhibitor-free, Honeywell) was dried over molecular sieves ($H_2O$ content <10 ppm) and mixed with 0.2 M $LiClO_4$ (99.99% trace metal basis, battery grade, Sigma-Aldrich). Lithium bis(oxalato)borate, LiBOB (Chemetall GmbH) and Ethylene Carbonate, EC (>99%, acid <10 ppm, $H_2O$, Sigma-Aldrich) were added to the base electrolyte to the electrolyte to achieve correct concentrations (5 volume-% EC and 50 mM LiBOB). Porous glassy carbon (GC) electrodes used in the IR and OEMS cells were prepared by mixing 95 wt% GC powder (spherical powder, 2–12 µm, 99.95% trace metals basis, Sigma-Aldrich) and 5 wt% PVDf (Kynar Flex 900 HSV, Arkema) binder in N-methyl-2-pyrrolidone (Sigma-Aldrich) solution. The solution was mixed for 30 min at 25 Hz (MM 400, Retsch). The slurry was coated on a stainless-steel mesh (212/90 µm, Bopp AG) with a 150 µm gap applicator. Carbon QCM-sensors were prepared by coating commercial 5 MHz Au type sensors (Quartz Pro Sweden AB) with first a 50 nm thick Cr adhesion film and then a 50 nm thick C layer by sputter deposition at room temperature. $LiFePO_4$ electrodes (1 mAh $cm^{-2}$, Custom Cells) were punched (θ 15 mm for the OEMS cell, θ 12 mm for the EQCM cell) and delithiated to 90% of initial charge (3.43 V versus $Li^+/Li$). All electrodes used were dried at 120 °C for 12 h in a vacuum oven before cell assembly.

FTIR absorbance spectra were recorded with a Bruker Vertex 70V equipped with a liquid $N_2$ cooled MCT detector. All spectra were recorded with 4 $cm^{-1}$ resolution over 128 scans. *Operando* IR spectra were recorded every 250 s and are presented as differential absorbance

spectra relative to the spectrum at open circuit potential (OCP). Before subtraction of the OCP spectrum, atmospheric $H_2O$ compensation and a baseline correction were performed on all *operando* spectra.

The electrochemical cells and the data treatment procedure for *operando* measurements have been described in previous publications[1–3] except for the *operando* IR cell. The cell setup used for these measurements, a similar setup with as Dillard et al. was used.[4] All electrode potentials presented relative to the $Li^+$/Li redox couple. Cyclic voltammetry with a sweep rate of 0.1 mV $s^{-1}$ (except for the IR measurements where a scan rate of 0.2 mV $s^{-1}$ was used) was performed from OCP to vertex potentials of 0.5 V and 1.7 V.

$^{11}$B-NMR spectrum was recorded on an Agilent 400 MHz NMR spectrometer (model Agilent MR400-DD2). Anhydrous dimethylsulfoxide (DMSO-$d_6$, 99.9%, VWR) was used as deuterated solvent.

## 2. Structure of Lithium bis(oxalato)borate

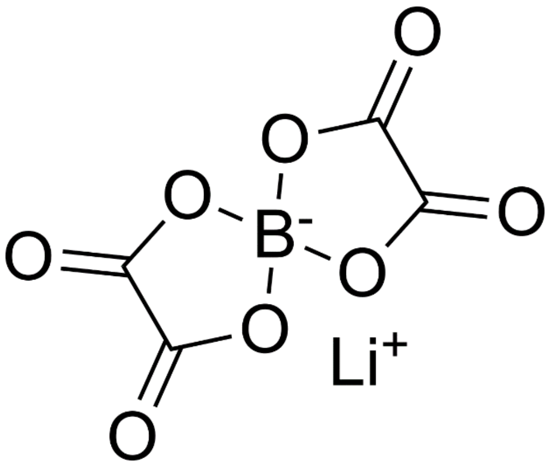


**Figure S1**. Structure of lithium bis(oxalato)borate, LiBOB.

## 3. $^{11}$B-NMR of LiBOB

The reduction process of LiBOB and the effects of contaminants from synthesis have been studied and discussed extensively ever since the salt was introduced and are often ascribed to be the cause for the reduction process observed at high potentials.[5,6] To ensure the purity of the LiBOB salt in this study, $^{11}$B-NMR was performed on LiBOB dissolved in DMSO (figure S 1). Only one strong signal around 7.3 ppm, assigned to LiBOB, was detected, i.e. no signs of any boron-containing species besides LiBOB.

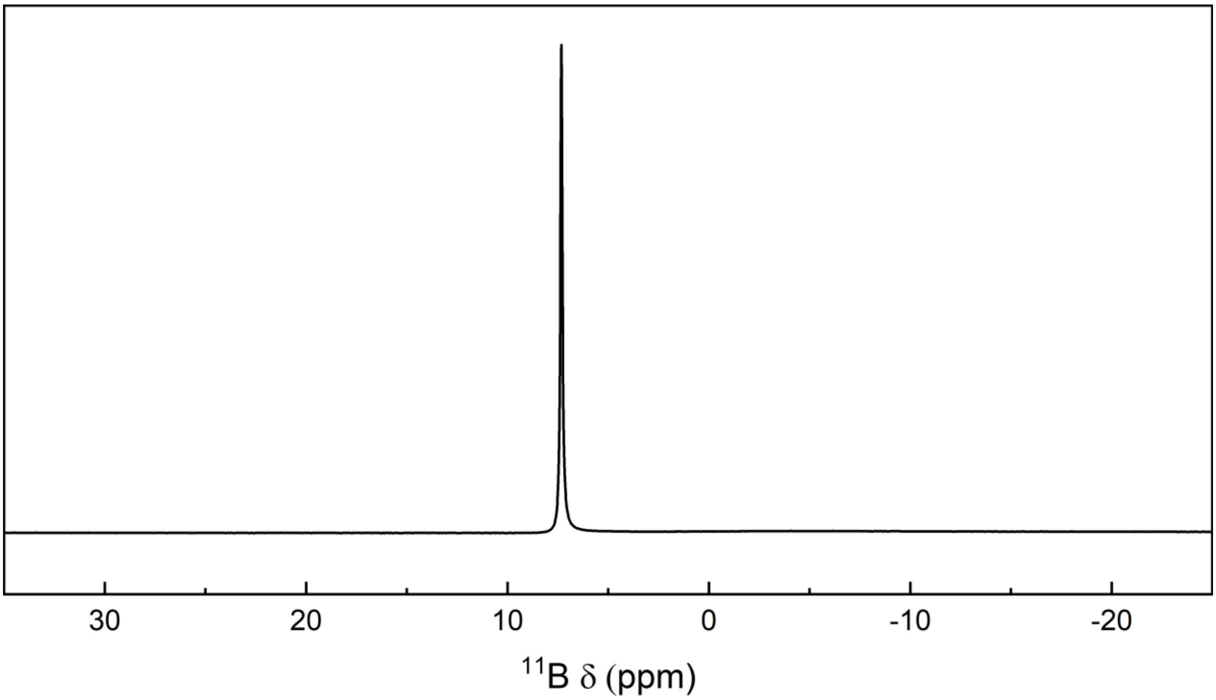


**Figure S2**. $^{11}$B-NMR spectra of LiBOB dissolved in DMSO-$d_6$.

## 3. Operando IR - 0.2 M $LiClO_4$ in DME 50 mM LiBOB

Figure S2 shows the uncorrected (i.e. not differential spectra relative to OCP spectrum) *operando* IR absorbance spectra of a porous GC electrode in 0.2 M $LiClO_4$ in DME + 50 mM LiBOB during an LSV from OCP to 0.5 V. At the bottom a spectrum of pure DME is included to demonstrate how the *operando* spectra are dominated by the vibration modes of DME.

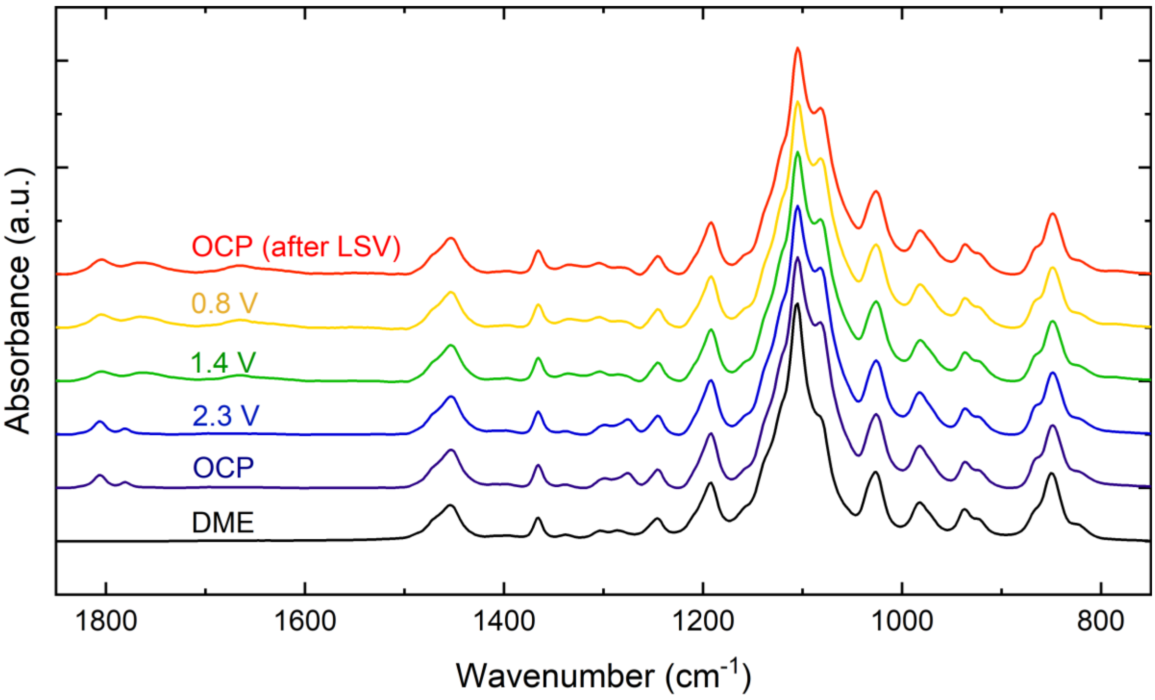


**Figure S3.** *Operando* IR absorbance spectra recorded during a linear sweep voltammogram of a porous GC electrode pressed on a diamond ATR crystal in 0.2 M $LiClO_4$ in DME + 50mM LiBOB and a spectrum of pure DME solvent.

## 4. Operando IR - 0.2 M $LiClO_4$ in DME+ 50 mM LiBOB + 5 vol% EC

Figure S3 shows the differential spectra (relative to OCP spectrum) *operando* IR absorbance spectra of a porous GC electrode in 0.2 M $LiClO_4$ in DME + 50 mM LiBOB + 5 vol% EC during an LSV from OCP to 0.5 V. Note that the vibration modes of $BOB^-$ are overlapped by the C=O vibration mode of EC.

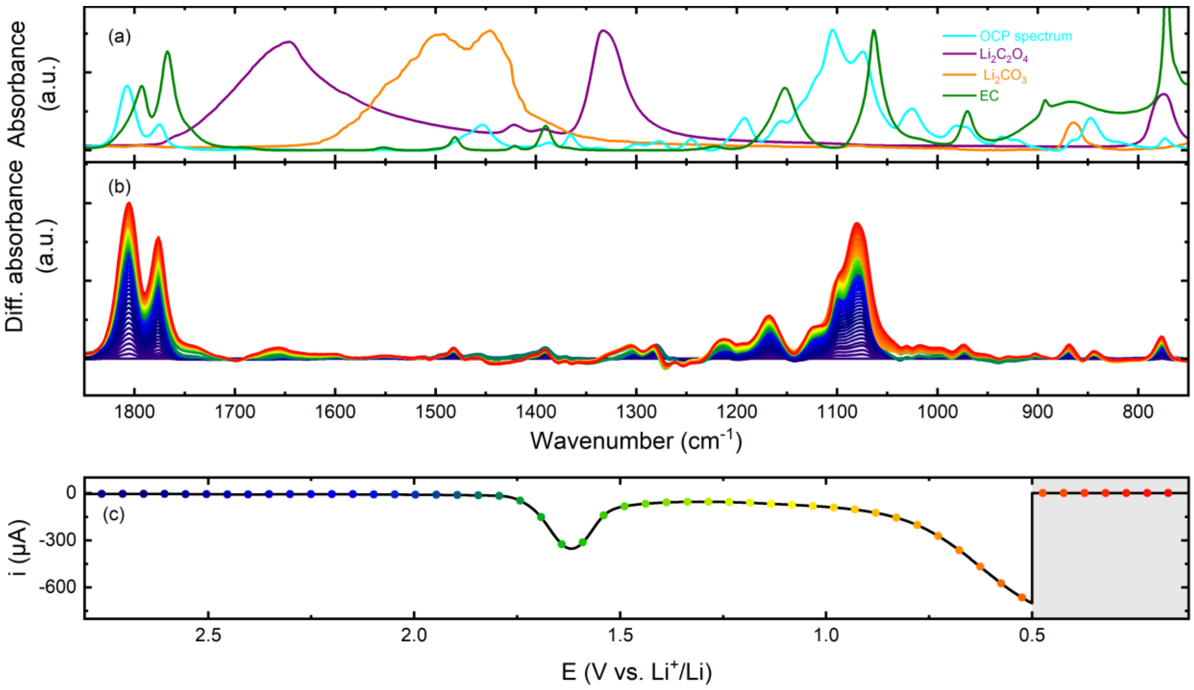


**Figure S4.** (a) Reference absorbance spectra of a porous GC soaked in 0.2 M $LiClO_4$ in DME + 50mM LiBOB + 5 vol% EC at OCP ("OCP spectrum" in figure), $Li_2C_2O_4$, $Li_2CO_3$ and EC, (b) *Operando* IR differential absorbance spectra relative to a spectrum at open circuit potential (OCP) with corresponding (c) linear sweep voltammogram of a porous GC electrode pressed on a diamond ATR crystal in 0.2 M LiClO4 in DME + 50mM LiBOB+ 5 vol% EC.

In this *operando* IR measurement, only positives features are present, which differs significantly from the spectra seen in figure 1 of GC electrode polarized in 0.2 M $LiClO_4$ in DME + 50mM LiBOB. The positive features resemble both the OCP spectrum, i.e. DME, and EC. Even though the reduction of $BOB^-$ is clearly present in the LSV (Fig. S3 (c)), only minor features at the wavenumbers of $Li_2C_2O_4$ is observed.

# References


(1) Lundström, R.; Berg, E. J. Design and Validation of an Online Partial and Total Pressure Measurement System for Li-Ion Cells. *J. Power Sources* **2021**, *485*, 229347.

(2) Kitz, P. G.; Lacey, M. J.; Novák, P.; Berg, E. J. Operando EQCM-D with Simultaneous in Situ EIS: New Insights into Interphase Formation in Li Ion Batteries. *Anal. Chem.* **2019**, *91*, 2296–2303.

(3) Kitz, P. G.; Novák, P.; Berg, E. J. Influence of Water Contamination on the SEI Formation in Li-Ion Cells: An Operando EQCM-D Study. *ACS Appl. Mater. Interfaces* **2020**, *12*, 15934–15942.

(4) Dillard, C.; Singh, A.; Kalra, V. Polysulfide Speciation and Electrolyte Interactions in Lithium-Sulfur Batteries with in Situ Infrared Spectroelectrochemistry. *J. Phys. Chem. C* **2018**, *122*, 18195–18203.

(5) Xu, K.; Zhang, S. S.; Lee, U.; Allen, J. L.; Jow, T. R. LiBOB: Is It an Alternative Salt for Lithium Ion Chemistry? *J. Power Sources* **2005**, *146*, 79–85.

(6) Yang, L.; Furczon, M. M.; Xiao, A.; Lucht, B. L.; Zhang, Z.; Abraham, D. P. Effect of Impurities and Moisture on Lithium Bisoxalatoborate (LiBOB) Electrolyte Performance in Lithium-Ion Cells. *J. Power Sources* **2010**, *195*, 1698–1705.
