# Supplementary material for: Elucidating the Reduction Mechanism of Lithium Bis(oxalato)borate
Source: J Phys Chem Lett. 2024 Feb 28;15(9):2537–41. doi: 10.1021/acs.jpclett.4c00328 (PMC10926156; doi:10.1021/acs.jpclett.4c00328)
Supplement: Supplementary file 2 — jz4c00328_si_002.pdf [file jz4c00328_si_002.pdf]

jz-2024-00328g.R1

Name: Peer Review Information for "Elucidating the Reduction Mechanism of Lithium bis(oxalato)borate"

First Round of Reviewer Comments

Reviewer: 1

Comments to the Author

The authors provide a detailed and systematic study on understanding the reduction mechanism of a common additive lithium bis(oxalato)borate (LiBOB) within a model electrolyte. The authors use 3 complementary methods: in situ infrared, EQCM and OEMS so that the solid and gaseous products are both monitored. The data is very compelling and shows LiC2O4 is the main product - from alignment with known IR bands and from EQCM mass per electron of ca. 50 g mol-1. OEMS shows concurrent appearance of CO2 with the reduction peak of LiBOB. The protective film forming properties of LiBOB in presence of EC are elegantly shown - with the additive layer preventing C2H4 formation.

Minor Comments:

Figure 4 referred to first. Authors may wish to address this by having LiBOB structure also in SI as Figure S1.

Color for Li2C2O4 and Li2CO3 too similar (on print out copy, ok online – but maybe something authors to consider).

Key text in Figure 1d too small.

Reviewer: 2

Comments to the Author

This is a very interesting manuscript which provides a detailed investigation of an important electrolyte additive for lithium ion batteries. The manuscript should be accepted with the following

minor revision. There have been numerous other investigations of LiBOB, the authors should provide additional description of the novelty and urgency of the manuscript.

Author's Response to Peer Review Comments:

jz-2024-00328g

Elucidating the Reduction Mechanism of Lithium bis(oxalato)borate

We are grateful to the reviewers for taking time to read and come with input on our manuscript. The comments from the reviewers are color coded (reviewer #1 in red and reviewer #2 in blue) with our response following directly after.

Reviewer: 1. The authors provide a detailed and systematic study on understanding the reduction mechanism of a common additive lithium bis(oxalato)borate (LiBOB) within a model electrolyte. The authors use 3 complementary methods: in situ infrared, EQCM and OEMS so that the solid and gaseous products are both monitored. The data is very compelling and shows LiC2O4 is the main product - from alignment with known IR bands and from EQCM mass per electron of ca. 50 g mol-1. OEMS shows concurrent appearance of CO2 with the reduction peak of LiBOB. The protective film forming properties of LiBOB in presence of EC are elegantly shown - with the additive layer preventing C2H4 formation.

Minor Comments:

Figure 4 referred to first. Authors may wish to address this by having LiBOB structure also in SI as Figure S1.

We agree with reviewer 1 in that referring to figure 4 is not correct. A figure of the structure of LiBOB has been added as figure S1 in the Supporting information as well as a reference to it in the introduction, along the reference to figure 4.

Color for Li2C2O4 and Li2CO3 too similar (on print out copy, ok online – but maybe something authors to consider).

The color indicating Li_2CO_3 has been changed to orange in all figures in the manuscript and supporting information.

Key text in Figure 1d too small.

The font size of the key text has been increased by 0.5 points. Making the key text larger than this is not possible due to the limited available space in figure 1d, unfortunately.

Reviewer: 2. This is a very interesting manuscript which provides a detailed investigation of an important electrolyte additive for lithium ion batteries. The manuscript should be accepted with the following minor revision. There have been numerous other investigations of LiBOB, the authors should provide additional description of the novelty and urgency of the manuscript.

To emphasize the novelty and urgency of this study, the following paragraph has been added to the conclusions in of the end of the manuscript;

In this study, complementary operando techniques are utilized to monitor solid, liquid, and gaseous reaction species during the reduction process of a crucial additive. By doing so, existing uncertainties surrounding the reduction mechanism of LiBOB are addressed. This study not only contributes to fundamental knowledge but also underscores the significance of advancing operando techniques. Furthermore, it demonstrates the value of revisiting wellstudied systems with novel experimental approaches.
